# New HIV-1 circulating recombinant form 94: from phylogenetic detection of a large transmission cluster to prevention in the age of geosocial-networking apps in France, 2013 to 2017

**DOI:** 10.2807/1560-7917.ES.2019.24.39.1800658

**Published:** 2019-09-26

**Authors:** Marc Wirden, Fabienne De Oliveira, Magali Bouvier-Alias, Sidonie Lambert-Niclot, Marie-Laure Chaix, Stéphanie Raymond, Ali Si-Mohammed, Chakib Alloui, Elisabeth André-Garnier, Pantxika Bellecave, Brice Malve, Audrey Mirand, Coralie Pallier, Jean-Dominique Poveda, Theresa Rabenja, Veronique Schneider, Anne Signori-Schmuck, Karl Stefic, Vincent Calvez, Diane Descamps, Jean-Christophe Plantier, Anne-Genevieve Marcelin, Benoit Visseaux, on behalf of the French National Agency for Research on AIDS and Viral Hepatitis (ANRS) AC43 Study Group

**Affiliations:** 1 Sorbonne Université, INSERM, Institut Pierre Louis d'Epidémiologie et de Santé Publique IPLESP, AP-HP, Hôpital Pitié Salpêtrière, Laboratoire de virologie, Paris, France; 2 Normandie Université, UNIROUEN, EA2656 GRAM, CHU de Rouen, Laboratoire de virologie associé au CNR VIH, Rouen, France; 3 HU Henri Mondor, Laboratoire de virologie, Créteil, France; 4 AP-HP, Hopital Saint-Antoine, Laboratoire de virologie, Paris, France; 5 AP-HP, Hôpital Saint-Louis, Laboratoire de virologie, INSERM U944, Paris, France; 6 Laboratoire de virologie, CHU Purpan de Toulouse, Toulouse, France; 7 Laboratoire de virologie CHU de Dijon, Dijon, France; 8 Laboratoire de virologie, Hôpital Avicenne, Bobigny, France; 9 Laboratoire de virologie, CHU Hôtel Dieu, Nantes, France; 10 Laboratoire de virologie CHU de Bordeaux, Bordeaux, France; 11 Laboratoire de virologie CHU de Nancy, Nancy, France; 12 Laboratoire de virologie CHU de Clermont-Ferrand, Clermont-Ferrand, France; 13 Laboratoire de virologie, Hôpital P. Brousse, Villejuif, France; 14 Laboratoire CERBA, Saint-Ouen-l'Aumône, France; 15 Laboratoire du Grand Hôpital de l’Est Francilien, Jossigny, France; 16 Laboratoire de virologie, Hôpital Tenon, Paris, France; 17 Laboratoire de virologie CHU de Grenoble, Grenoble, France; 18 Laboratoire de virologie CHU de Tours, Tours, France; 19 Laboratoire de virologie, AP-HP, Hopital Bichat Claude Bernard, Univ Paris-Diderot, INSERM, IAME, CNR VIH, Paris, France; 20 Members are listed in the acknowledgements

**Keywords:** HIV cluster, HIV CRF94, phylogenetic analysis, HIV Outbreak, HIV Prevention

## Abstract

**Background:**

Ending the HIV pandemic must involve new tools to rapidly identify and control local outbreaks and prevent the emergence of recombinant strains with epidemiological advantages.

**Aim:**

This observational study aimed to investigate in France a cluster of HIV-1 cases related to a new circulating recombinant form (CRF). The confirmation this CRF’s novelty as well as measures to control its spread are presented.

**Methods:**

Phylogenetic analyses of HIV sequences routinely generated for drug resistance genotyping before 2018 in French laboratories were employed to detect the transmission chain. The CRF involved was characterised by almost full-length viral sequencing for six cases. Cases’ clinical data were reviewed. Where possible, epidemiological information was collected with a questionnaire.

**Results:**

The transmission cluster comprised 49 cases, mostly diagnosed in 2016–2017 (n = 37). All were infected with a new CRF, CRF94_cpx. The molecular proximity of this CRF to X4 strains and the high median viraemia, exceeding 5.0 log_10_ copies/mL, at diagnosis, even in chronic infection, raise concerns of enhanced virulence. Overall, 41 cases were diagnosed in the Ile-de-France region and 45 were men who have sex with men. Among 24 cases with available information, 20 reported finding partners through a geosocial networking app. Prevention activities in the area and population affected were undertaken.

**Conclusion:**

We advocate the systematic use of routinely generated HIV molecular data by a dedicated reactive network, to improve and accelerate targeted prevention interventions. Geosocial networking apps can play a role in the spread of outbreaks, but could also deliver local targeted preventive alerts.

## Introduction

In 2014, a ‘90-90-90’ target was proposed by UNAIDS to help end the global acquired immunodeficiency syndrome (AIDS) epidemic by 2020 [1,2]. According to this target, 90% of infected people must be diagnosed, 90% of diagnosed people must be on antiretroviral treatment, and 90% of treated patients must display viral suppression. In countries of western Europe, highly efficient antiretroviral drugs, easy access to care, and the ‘treat all’ approach have made it possible to attain or get close to the last two of these objectives. Nevertheless, the incidence of HIV-1 infection has not decreased sufficiently, particularly among men who have sex with men (MSM) [3,4]. Screening for infection and upstream prevention are the weak links in the chain. In France, self-testing kits for HIV infection are now available over-the-counter and pre-exposure prophylaxis (PrEP) is supported by the healthcare system [5-7]. 

Recent advances in molecular epidemiology could facilitate the identification of transmission clusters and outbreaks, as well as the initiation of rapid and focused preventive activities to limit HIV spread, but their routine use is not easy [8-12]. Furthermore, the sustained HIV epidemic may allow new circulating recombinant forms (CRF) to emerge, particularly if different HIV subtypes are present, as in Europe [10,13-16]. Such genetic recombination may lead to the selection of more virulent HIV strains in the infected population. This study, reporting the discovery of a recent highly active transmission cluster, with factors boosting the spread of a new HIV recombinant, illustrates all these points. 

The objectives of this work were to confirm the novelty of the recombinant and to estimate the extent of this cluster in France, as well as its origins and transmission pathways. Targeted prevention activities guided by results of phylogenetic and epidemiological data analyses to control the spread of this new HIV-1 strain were attempted.

## Methods

### Detection of a transmission cluster

Between 2015 and 2017, the Pitié Salpêtrière Hospital laboratory received 10 plasma samples for routine HIV-1 resistance genotyping — recommended at time of diagnosis — harbouring virus strains with identical subtyping discordances on analysis with the Los Alamos laboratory subtyping tools (https://www.hiv.lanl.gov/content/sequence/BASIC_BLAST/basic_blast.html). While the protease (PR) and integrase (INT) genes in the sequences derived from those samples, exhibited perfect matches with the CRF02_AG references, the reverse transcriptase (RT) gene matched the subtype B references. Moreover, the Basic Local Alignment Search Tool (BLAST) revealed high levels of similarity between the 10 strains: 100% for PR nt sequences, and 100 to 99.6% for the RT and INT sequences. These observations were suggestive of an active transmission cluster involving a new CRF of HIV-1 combining both the subtype B, the major HIV lineage in countries of western Europe but absent from Africa, and the CRF02_AG associating subtypes A and G, one of the oldest HIV recombinant lineage, which is mostly present in West Africa and is the main non-B lineage in France.

### Study population

In September 2017, to verify the hypothesis of a cluster related to a new CRF, a prototype set of sequences was sent to all laboratories participating to the *Agence Nationale de Recherche sur le SIDA et les Hépatites Virales* (ANRS) AC43 resistance study group, nationwide, for testing and identification of similar strains. All available HIV-1 nt sequences displaying the same subtyping discordances as the reference set were collected, prospectively and retrospectively, until December 2017. Patients were included in the study if these similarities were confirmed by phylogenetic analyses, as described below.

Clinical data, sex, age, together with HIV-1 viral loads and CD4 cell counts, were collected at diagnosis and after treatment initiation. Where possible, epidemiological information was collected with an anonymous questionnaire and the patient’s consent. The questions included the date of the last HIV negative test, the probable transmission pathway, the place of residence and where infection probably occurred, the circumstances of dating (casual or regular partner or stranger, with dating apps, or cruising area or other), and the knowledge of PrEP. Because a specific company with clearly identified activities had been frequently and spontaneously reported by the first patients, we also asked if the patients, or their partners, or the place where infection may have occurred, had a link with any company with such activities in the suburbs of Paris.

### Ethical statement

The study was based on routine patient follow-up biological data and did not involve any additional sampling. Ethical approval was not needed for this study.

### HIV resistance genotyping and tropism determination

In the ANRS AC43 study group laboratories, the PR, RT, INT and V3 *env* genes had been routinely sequenced with the ViroSeq HIV-1 Genotyping System (Applied Biosystems, Foster City, California (CA)), and/or the ANRS method (http://www.hivfrenchresistance.org), depending on the laboratory and the amplification failures encountered. HIV tropism, C-C chemokine receptors type 5, CCR5 (R5) or C-X-C chemokine receptors type 4, CXCR4 (X4), had been determined with V3-loop sequencing and the Geno2pheno algorithm, with a false-positive rate (FPR) threshold of 10% (https://coreceptor.geno2pheno.org/). A recombinant virus phenotypic entry test result was also available for three patients [17].

### Phylogenetic and epidemiological investigation of the new circulating recombinant form cluster

For confirmation that all the collected viral strains belonged to the same cluster, the PR + RT and INT sequences were aligned with all the HIV-1 group M subtype and CRF reference sequences from the Los Alamos National laboratory database (http://www.hiv-web.lanl.gov; n = 181). The seven previously described unique recombinant forms including both subtype B and CRF02_AG sequences were also included in the comparison [18]. Phylogenetic trees were constructed by maximum likelihood methods with IQ Tree 1.6.1 under the General Time Reversible with Gamma distribution of rates across sites (GTR-G) nt substitution model, with ultrafast bootstrapping and 1,000 replicates.

This phylogenetic analysis was combined with temporal and geographical information to improve the characterisation of transmission within this cluster.

### Analysis of the new circulating recombinant form

Sanger sequencing of seven overlapping fragments, as previously described, enabled to recover near-to-complete genome sequences for six plasma samples [19]. The samples were respectively obtained from six patients who were not directly linked epidemiologically and belonged to the four main sub-clusters of the PR + RT phylogenetic tree.

The six sequences were aligned with all full-length genome group M reference sequences of the Los Alamos database, for determination of the recombination breakpoints. This alignment was analysed with the Recombination Detection Programme (RDP) version 4.95. For each fragment identified, the corresponding lineages were confirmed by maximum likelihood phylogenetic reconstruction with IQ Tree 1.6.1, as described above. The six near-complete genome sequences were deposited in the GenBank database (accession numbers MH141491 to MH141494 and MH683549, MH683550).

To check the novelty of the identified recombination pattern, it was compared with all previously described CRFs and, more particularly to the CRF56_cpx, the only one containing both subtype B and CRF02_AG ancestors. All previously described unique recombinant forms (URF) containing both CRF02_AG and subtype B ancestors were also extensively retrieved from the scientific literature to verify any potential previous identification [13,18].

## Results

### Patient characteristics

**Figure 1:**
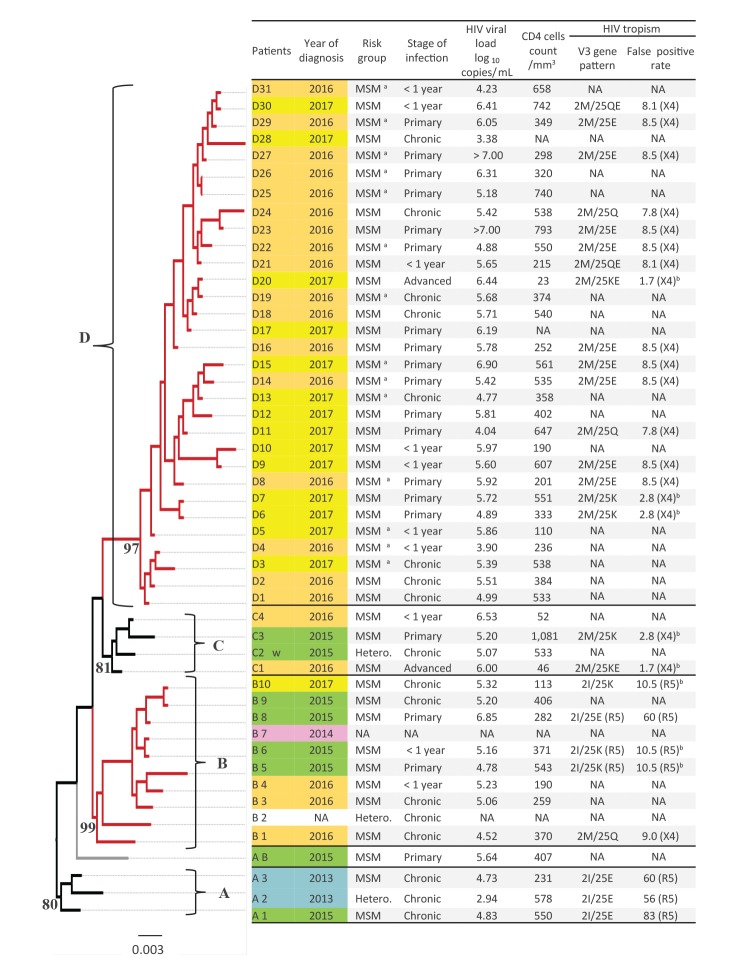
Phylogenetic tree obtained from the sequences of the HIV-1 protease plus reverse transcriptase regions, which were collected during the investigation of a transmission cluster, France, 2013–2017 (n = 49)

A total of 49 patients were included in the study (Figure 1). Their infection was diagnosed in 2013 (n = 2), 2014 (n = 1), 2015 (n = 8), 2016 (n = 22), and 2017 (n = 15), with year of diagnosis unknown for one patient. All but one of the patients were male, and all were Caucasians of French origin. Median age was 35 years (interquartile range (IQR): 28–43 years), and 41 of the 49 patients were diagnosed in the Ile-de-France region. The transmission route was MSM for 45 patients, heterosexual for three, and unknown for one. The reasons for HIV testing were clinical symptoms of primary infection (n = 19), systematic HIV testing (n = 12), other sexually transmitted infections (n = 8), regular partner testing positive for HIV (n = 3), or not reported (n = 7). HIV-1 infection was recent in 29 patients, including 19 with primary infection and 10 with a negative test result within the preceding year. In the 24 cases for which information was available, risky sexual behaviour was reported during the probable infection period, with strangers (20/24), and/or acquaintances (12/24), and/or a regular partner (5/24). Casual partners were mostly encountered through a geosocial networking app (20/24), and/or in gay cruising areas (7/24). None of the patients was on PrEP, and 18/24 were unaware of this preventive method at the time of diagnosis.

Hetero.: heterosexual; MSM: men who have sex with men; NA: not available; w: women.

^a^ Direct or indirect link to a specific company.

^b^ The 11/25 rule would predict an X4-strain.

Bootstrap values ≥ 80% defined four sub-clusters, A, B, C and D. Colours (reused in Figure 3) indicate the same year of diagnosis. Some parameters at the time of diagnosis are reported. The amino acids in positions 2 and 25 of the V3 protein are shown, when known.

In the last column, the false positive rate and the tropism in parentheses are depicted according to the output of the Geno2pheno algorithm (with a threshold of 10%) which reported also the result of the 11/25 rule when this one predicted an X4 tropism (footnote b).

The scale bar indicates the length of horizontal branches corresponding to a genetic distance of 0.003 substitution per nucleotides.

### Biological parameters and treatment outcomes

For non-primary and primary infections, respectively, the median HIV-1 load at diagnosis was 5.28 (IQR: 4.80–5.89) and 5.78 (IQR: 5.19–6.28) log_10_ copies/mL, and the median CD4 cell count 371 (IQR: 196–539) and 407 (IQR: 303–558) /mm^3^. No drug resistance mutations other than those in the genetic sequence leading to the classical CRF02_AG polymorphic L10I, K20I and M36I mutations of the PR protein were identified among the 49 samples. The ViroSeq genotyping system, which is routinely used in many laboratories, failed to amplify the PR and RT genes in seven of 11 cases, even with viraemia above 5.0 log_10_ copies/mL. In these cases, the ANRS method was used as second test to obtain the data.

HIV tropism was determined for 26 patients. V3 amino-acid (AA) sequences displayed five different patterns, according to the residues in positions 2 and 25. These two positions drove major FPR changes, leading to a change in tropism from R5 strains in patients diagnosed in 2013, to X4 strains in those diagnosed in 2016 and 2017 (Figure 1). The two patients (C1 and D20) diagnosed at an advanced stage with fewer than 50 CD4 + T-cells/mm^3^, harboured a mixture of the two X4 patterns (2M/25K and 2M/25E). The recombinant phenotype tests yielded conflicting results relative to those of the Geno2pheno algorithm, with dual R5/X4 usage for D20 who harboured the two X4 patterns and only R5 coreceptor usage for patients C3 (2M/25K) and D27 (2M/25E).

HIV viral loads after 6 and 12 months of treatment were available for 36 and 31 patients, respectively. Before treatment, median viraemia was 5.42 log_10_ copies/mL (IQR: 5.04–5.94), and CD4 cell count was 371/mm^3^ (IQR: 235–550). The first combined antiretroviral therapy (cART) received systematically consisted of two nucleoside reverse transcriptase inhibitors (NRTIs), plus an integrase inhibitor (for 21 of the 36 patients), a protease inhibitor (13 of 36 patients), or a non-NRTI (2 of 36 patients). After 6 and 12 months of treatment, 27 and 25 of these patients, respectively, had a viraemia below 50 copies/mL, with median CD4 counts of 634/mm^3^ (IQR: 478–950) and 777/mm^3^ (IQR: 498–900), respectively. Thus, six of 31 patients did not reach the therapeutic goal after 12 months on cART, but their viraemia was < 100 copies/mL except for patient D20 (550 copies/mL and without resistance mutations). Before treatment, the median viraemia and CD4 cell count of these six individuals were 5.82 log_10_ copies/mL and 478/mm^3^, respectively. Only two of them were in primary infection at baseline.

### Characteristics of the new circulating recombinant form 

**Figure 2:**
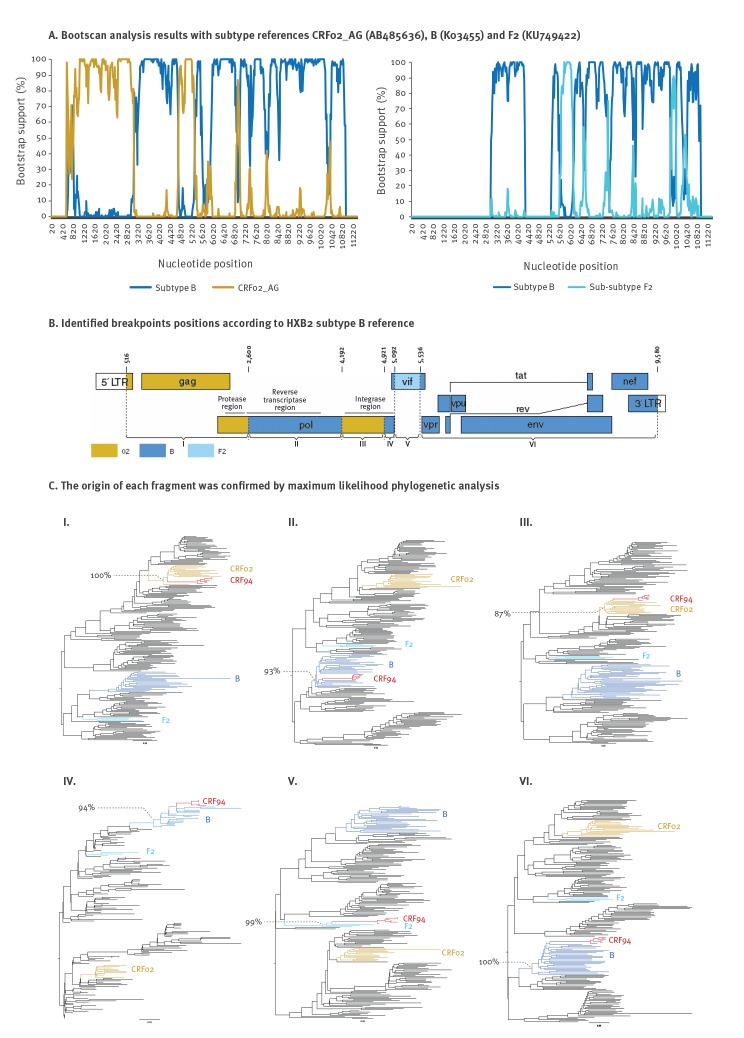
Characterisation of the circulating recombinant form 94 composed of subtype B, CRF02_AG and F2 fragments with recombination breakpoints, France, 2013–2017

LTR: long terminal repeat; vif: virion infectivity factor; vpr: virion-associated protein; vpu: viral protein U.

The full-length genome analysis, confirmed the discovery of a new CRF, CRF94_cpx, composed of subtype B, CRF02_AG and F2 segments (Figure 2). All B/CRF02_AG identified recombination breakpoints were identified with more than six of the nine methods included in RDP 4.95. The F2 fragment was identified only with RDP and bootscan methods. However, the Los Alamos BLAST tools and the phylogenetic trees reconstructed for the corresponding genome fragment confirmed the strong sequence similarity between the CRF94_cpx strains and the F2 lineage. This CRF is the first containing a fragment of this rare sub-subtype F2 (belonging to subtype F), originally described in Cameroon according to the Los Alamos laboratory database (https://www.hiv.lanl.gov/components/sequence/HIV/search/search.html), to be reported.

The percentages on the trees presented in panel 2C indicate the branch support of the node including the CRF94 sequences with the corresponding HIV subtype (ultrafast bootstrap).

### Transmission cluster evolution in the context of geographical data

**Figure 3:**
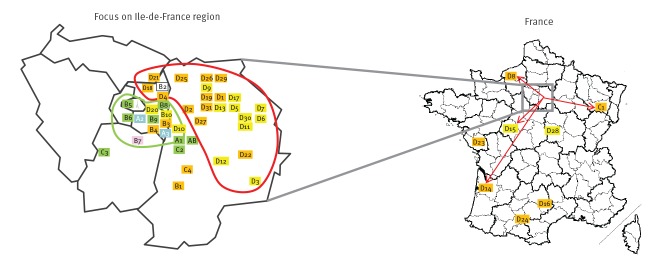
Geographical distribution of residence for the patients described in Figure 1 who were involved in a HIV transmission cluster, France, 2013–2017 (n = 49)

The PR + RT phylogenetic analysis showed that all strains formed a single recent transmission cluster (RTC) defined as a monophyletic clade with a branch support value > 95% (98%) and a maximum genetic distance < 4.5% (3.4%). Based on an analysis of time to most recent common ancestor (tMRCA), this RTC was estimated to have emerged in July 2012. In the phylogenetic tree, this RTC is divided into four distinct sub-clusters named A, B, C and D, with respective nodes supported by bootstrap values ≥ 80% (Figure 1). Interestingly, the patients diagnosed in 2013–2015 were all included in the first three sub-clusters (A–C), whereas the last sub-cluster (D) harboured only the patients diagnosed in 2016–2017. Moreover, the sub-clusters also had different geographical distributions (Figure 3), as patients from sub-clusters A and B resided in an area centred on the Val-de-Marne, whereas the patients in sub-cluster D were mostly from the north of the Seine-et-Marne or more distant French regions. We found that at least 14 of the 31 patients in sub-cluster D had links to a single company in north of Seine-et-Marne, having worked for this company or engaged in risky sexual behaviour with employees of the company during the likely period of their infection. 

Different colours indicate different years of diagnosis: blue for 2013, pink for 2014, green for 2015, orange for 2016 and yellow for 2017. The green and red circles show the geographical shift of the outbreak between 2013 and 2016–17. The red arrows indicate a displacement of individuals before their diagnosis.

Eight patients were diagnosed outside Ile-de-France. One (C1) of these had moved from the area concerned, and three others (D8, D14 and D15) were seasonal workers in the company in the month preceding primary infection (Figure 3). No information was available for the four other individuals. 

These findings made it possible to identify a precise area and population playing a major role in this outbreak and its sudden acceleration since 2016.

### Outbreak control measures

French preventive care organisations, and local healthcare organisations in the north of Seine-et-Marne, as well as associations involved in HIV prevention were informed of our observations. The local organisations were already aware of the existence of a large gay community working for this company and living in this area, displaying high-risk sexual behaviours. Preventive activities had been implemented within this population in the past, but had been stopped for several years. The discovery of this very active transmission cluster was the trigger for new targeted preventive activities in and around the company, and the places of residence of its staff, as well as in the gay cruising areas known by the local associations. These activities began in 2018 and consisted of HIV screening days, the promotion of condom use, as well as the distribution of information about post- and pre-exposure prophylaxis. As commonly done, the HIV screening days were broadcasted through one of the gay dating apps, but without any specific warning regarding this outbreak.

## Discussion

We show here that, even in a setting in which HIV prevention and treatments are widely available, important HIV transmission chains can still emerge. These transmission chains may lead to the selection of new recombinant strains with a potentially greater capacity to spread, and new tools are required to detect them early and to optimise prevention of their spread.

The cluster identified was exceptional in its size, comprising 49 patients, two-thirds of whom were diagnosed, and probably infected, within a two-year period (2016–17). A recent retrospective study including only patients infected for less than 6 months but diagnosed over a period of 15 years, resulted in the finding of 44 French clusters, with a median of only four individuals (range: 3–41) per cluster [10]. The real number of people involved in the CRF94_cpx outbreak is undoubtedly higher, as some infected individuals may not yet have been tested for HIV, or may have been diagnosed outside the French ANRS network. This may apply particularly to foreign seasonal workers, who are frequently hired by the company concerned and who subsequently return to their home countries. The large size of this cluster may reflect a large number of MSM with risky sexual behaviour within a limited geographical area, particular characteristics of the new emerging recombinant strain, or both.

As frequently reported for HIV transmission clusters, most of the patients identified were MSM, and the proportion of primary and recent HIV infections was high [10]. The high levels of HIV viraemia observed in the patients with primary infections in this cohort were similar to those reported in comparable cohorts, but the patients diagnosed during chronic infection had a higher median viral load: 5.3 log_10_ (IQR: 4.8–5.9), vs only 4.6 (IQR: 4.0–5.1) log_10_ copies/mL (p < 0.0001, data not shown) reported in the same ANRS network [20]. Normally, the first antiretroviral regimen leads to HIV RNA suppression before 6 months [21]. The high level of viraemia before treatment initiation may account for the inability to achieve this goal in six of 31 patients even after 12 months of treatment. The higher levels of replication in these patients may be a consequence of CRF94_cpx genetic recombination [22], increasing the risk of transmission of strains with an epidemiological advantage, capable of rapid spread.

The switch from R5 to X4 tropism over time between the A/B and C/D sub-clusters is also concerning. In cases of infection via sexual intercourse, R5 viruses generally predominate, accounting for more than 90% of cases, particularly in contexts of transmission clusters and primary infections [23-26]. X4 viruses are more prevalent in untreated patients at late stages of disease, with immunodeficiency [27,28]. In this cluster, most of the patients, including those in sub-cluster D in particular, were diagnosed with primary or recent infections. The FPR threshold of 10% must be sufficiently specific for the identification of X4 strains, but even with a more conservative 5% threshold, 5/26 patients harboured an X4 virus. The major change in the Geno2pheno tropism result is caused by the modification of residues at only two positions in V3-loop sequences. This small genetic difference clearly shows that CRF94_cpx is genetically close to both R5 and X4 viruses. This may explain the discordant results between Geno2Pheno tool predicting the strain to be X4 and phenotypic recombinant assays indicating R5 coreceptor usage for the 2M/25E pattern, which was observed in almost all branch D patients. This strain may have emerged during the outbreak in a patient at an advanced stage of immunodeficiency harbouring diversified X4 and R5 variants, like patient D20, for example. This hypothesis is consistent with recent suggestions that, in a context of transmission chains, the transmission bottleneck results in the selection of strains displaying R5 tropism [24]. X4 viruses are associated with a higher risk of progression to clinical AIDS [29]. The genetic proximity of CRF94_cpx to such strains is, thus, a matter of concern. Given the high levels of viraemia, facilitating transmission, and the difficulties decreasing viral replication to undetectable levels on treatment, these observations highlight the need to diagnose and treat individuals infected with this recombinant rapidly, to prevent further infection.

The high replication rate of this strain may not be the only characteristic favouring its spread. CRF94_cpx spread slowly through the southern suburbs of Paris in 2013–2014 (Figure 3, sub-clusters A, B and C). When the CRF94_cpx outbreak reached the eastern suburbs of Paris (sub-cluster D), the rate of infection increased due to faster spread through the population of MSM living or working in this area. Thus, the geographical concentration of MSM with risky behaviour was probably one of the factors promoting the spread of this particular strain. The well-localised nature of the outbreak can be explained by the proximity of the patients' workplace to their accommodation in nearby residences. 

The use of geosocial networking apps may also have favoured the spread of this strain. In this study, the vast majority of cases used such apps to identify sexual partners nearby, leading to the infection of other inhabitants of the area without links to the company concerned. Conversely, French seasonal workers not from this suburb were diagnosed after returning home. This would also be the case for foreign seasonal workers, who might be diagnosed later. These findings highlight the importance of acting rapidly at the source of the outbreak to prevent its spread, and of surveys of the emergence of this strain in other European countries. The identification of this CRF94-cpx in the Los Alamos National Laboratory HIV database and the publication of this study could contribute to this.

This work also suggests that better use of new tools could help to end HIV outbreaks. Most of the patients of the CRF94_cpx cluster were unaware of PrEP at diagnosis. Condom use remains the cornerstone of HIV prevention, but awareness of the existence of PrEP should be increased in at-risk populations, and PrEP prescription should probably be increased to a larger population, as recently suggested [30]. A national information campaign was launched in the 2018 summer by the French AIDES association. The widespread use of apps to have a date with casual sex partners must also be taken into account when preventing and controlling outbreaks [31]. App users are more difficult to contact through traditional physical means, so collaboration with application editors may be required. These tools are sometimes used to encourage screening tests, but their geotargeting aspects could also be used to send focused alerts in cases of active outbreaks. More targeted actions are likely to be more effective. As shown here, combinations of phylogenetic and epidemiological analyses can help to identify the areas and populations involved in outbreaks.

One of the limitations of this study is that targeted activities began late in this outbreak, in 2018, partly due to the fortuitous discovery of this transmission chain and the improvised nature of the data collection and analyses. Moreover, the effectiveness of implemented prevention activities will be also difficult to assess without the possibility to compare the outbreak evolution without such activities. Another limitation is the unknown number of patients infected with CRF94_cpx strains and involved in this transmission cluster, because they were not recognised or not diagnosed. It was impossible to search these patients in all laboratories throughout France and Europe.

In conclusion, routinely generated HIV sequence data and epidemiological information for newly diagnosed patients can be used to focus preventive activities to limit HIV spreading. For optimal efficiency, data collection and analysis must be more systematic, with modern tools and connected epidemiological and interventional frameworks. A coordinated network of professionals, including virologists, clinicians and actors in local prevention (professional or associative) should be involved and supported by regional health authorities. In France, discussions about the reorganisation of existing national epidemiological surveillance HIV networks are underway, with a view to incorporating these new possibilities.
